# Distribution of Wild Mammal Assemblages along an Urban–Rural–Forest Landscape Gradient in Warm-Temperate East Asia

**DOI:** 10.1371/journal.pone.0065464

**Published:** 2013-05-31

**Authors:** Masayuki Saito, Fumito Koike

**Affiliations:** Graduate School of Environment and Information Sciences, Yokohama National University, Yokohama, Japan; Liverpool John Moores University, United Kingdom

## Abstract

Urbanization may alter mammal assemblages via habitat loss, food subsidies, and other factors related to human activities. The general distribution patterns of wild mammal assemblages along urban–rural–forest landscape gradients have not been studied, although many studies have focused on a single species or taxon, such as rodents. We quantitatively evaluated the effects of the urban–rural–forest gradient and spatial scale on the distributions of large and mid-sized mammals in the world's largest metropolitan area in warm-temperate Asia using nonspecific camera-trapping along two linear transects spanning from the urban zone in the Tokyo metropolitan area to surrounding rural and forest landscapes. Many large and mid-sized species generally decreased from forest landscapes to urban cores, although some species preferred anthropogenic landscapes. Sika deer (*Cervus nippon*), Reeves' muntjac (*Muntiacus reevesi*), Japanese macaque (*Macaca fuscata*), Japanese squirrel (*Sciurus lis*), Japanese marten (*Martes melampus*), Japanese badger (*Meles anakuma*), and wild boar (*Sus scrofa*) generally dominated the mammal assemblage of the forest landscape. Raccoon (*Procyon lotor*), raccoon dog (*Nyctereutes procyonoides*), and Japanese hare (*Lepus brachyurus*) dominated the mammal assemblage in the intermediate zone (i.e., rural and suburban landscape). Cats (feral and free-roaming housecats; *Felis catus*) were common in the urban assemblage. The key spatial scales for forest species were more than 4000-m radius, indicating that conservation and management plans for these mammal assemblages should be considered on large spatial scales. However, small green spaces will also be important for mammal conservation in the urban landscape, because an indigenous omnivore (raccoon dog) had a smaller key spatial scale (500-m radius) than those of forest mammals. Urbanization was generally the most important factor in the distributions of mammals, and it is necessary to consider the spatial scale of management according to the degree of urbanization.

## Introduction

Wild mammals contribute to human well-being. For example, watching wild birds and mammals is a popular ecotourism activity for the general public [1], [2]. Even in urban landscapes, people seek mammal-related activities, such as feeding and watching wild squirrels [3], [4]. Observation of wildlife by urban residents improves their recognition and support of biodiversity conservation [5]. In California, USA, people who had observed kit foxes (*Vulpes macrotis mutica*) in urban environments were more likely to favor conservation not only of urban foxes but also those in natural habitats [6]. Likewise, urban residents of Japan expressed their desire to coexist with well-recognized mammals such as squirrels and hares [7], [8]. Information on mammal assemblages around urban areas is important for the conservation of mammals both within and outside urban areas.

Urbanization often decreases biodiversity and alters animal assemblages via habitat loss and fragmentation [9]–[14], food subsidies such as crops and garbage [15], [16], and human avoidance behavior [17]. However, species richness does not always decrease along an environmental gradient from urban to rural and may exhibit various patterns [18]. For example, species richness increases at intermediate levels of urbanization in some birds [19], butterflies [20], and lizards [21]. Species richness of carabids in urban and rural landscapes are higher than in suburban landscape due to the greater numbers of species that prefer forest and open habitats [22]. To better understand diversity patterns, it is necessary to clarify the habitat preferences of various species belonging to the regional fauna.

Reim et al. [23] studied various mammal occurrences in habitats along an urban gradient using several types of pre-baited traps. However, few studies have examined the changes in total mammal assemblages along urban–rural–forest gradients, except for gathering qualitative information [24], [25] or data on limited taxa, such as rodents [26], [27], and carnivores [28], [29]. To clarify the general pattern of mammal assemblages along urban–rural–forest gradients, more quantitative research on both the various environments and the occurrence of mammals in those environments is necessary [24], [30].

The changes in mammal communities along urban–rural–forest gradients might differ in various regions around the world. For example, red foxes (*Vulpes vulpes*) are common in urban areas in cool-temperate zones, both in London, England [31], and Sapporo, Japan [32], whereas red foxes are rare and limited to forest areas in the warm-temperate zone of Japan [25]. Although many studies of urban mammals have been conducted in North America, Europe, and Australia, only a few such studies have been performed in Asia [33]. If the mammal communities in Asia have the same tendency as those in other regions, then only mammals that are food and habitat generalists can adapt to urbanization [34]. Because urbanization is rapidly expanding in Asia, which supports much of the Earth's biodiversity [35], it is important to conduct mammal studies in Asian metropolitan regions.

In this study, we quantitatively evaluated the effects of the urban–rural–forest gradient on the distributions of wild mammal assemblages in the Tokyo metropolitan area of Japan. During the late 20th century, large forest and agricultural areas disappeared in Japan due to rapid urban development [36], causing a loss of biodiversity in Japanese cities (e.g., birds [37], butterflies [38], carabids [39], and ectomycorrhizal fungi [40]). Tokyo, which had a population of 37.2 million in 2011, is the most populous urban agglomeration in the world [41]. The population of Tokyo is projected to increase until at least 2025 (to 38.7 million people) [41], in spite of estimates that the entire population of Japan will decrease [42]. Due to urban sprawl, the Tokyo metropolitan area has an urban gradient from the center of the city to forest landscapes, making this area suitable for the quantification of the effects of urbanization on wild mammals. Our quantitative habitat models for a broad range of mammal species will help to reveal the effects of urbanization on mammal assemblages in Asian cities, which, in turn, will aid in the conservation of wild mammals through landscape planning.

We also clarified the key spatial scale for the distributions of various mammals. The spatial scale at which a mammal responds to environmental factors differs among species due to differences in home range sizes and other factors. However, little information is available on the key spatial scales for mammal species [43]. Identifying the key spatial scales and devising quantitative habitat models will improve the management of mammal assemblages through landscape planning [44]–[46].

## Materials and Methods

### Study area

The Tokyo metropolitan area is in the warm-temperate climatic zone (mean annual temperature and annual precipitation; 15.6°C and 1700 mm at Katsuura on the Boso Peninsula; 15.9°C and 1467 mm at Yokohama in the Tama Hills). The potential natural vegetation is evergreen broadleaf forest [47].

**Figure 1:**
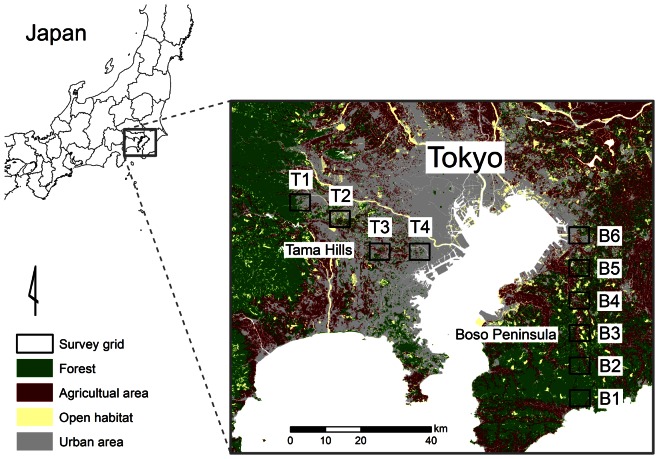
Map of the study area in the Tokyo metropolitan area of Japan. Survey grids were set in the Tama Hills (T1–T4) and on the Boso Peninsula (B1–B6), and 12 to 16 camera-trapping sites were placed in each grid.

We studied mammal assemblages along two transect lines at opposite sides of the metropolitan area, one on the Boso Peninsula and the other in the Tama Hills (Figure 1). Both areas are hilly, with elevations ranging from 0 to 408 m above sea level on the Boso Peninsula and from 50 to 200 m in the Tama Hills. The remnant forest fragments are abandoned coppiced forests (*Quercus serrata*) or cedar plantations (*Cryptomeria japonica*) on steep slopes. Flat lands are residential or agricultural areas, depending on the distance from the center of the metropolis. The Tama Hills are closer to the center of Tokyo and therefore have fewer agricultural areas (e.g., paddy fields) than the Boso Peninsula

Permission to carry out the field surveys was issued by the administrative offices of Uchiura-yama Forest Park, Ichihara Citizens' Forest, Nagaike Park, Oyamadairi Park, Oyamada Park, Shikinomori Park, and Mitsuike Park; the Ichihara Municipal Board of Education; the Chiba City Government; the Yokohama City Government; and the Tokyo Metropolitan Government.

### Field survey

We established six survey grids on the Boso Peninsula and four survey grids of the same size in the Tama Hills (Figure 1). Each survey grid was 3′45″ longitude by 2′30″ latitude (nearly 5 km by 5 km). To evaluate the occurrence of wild terrestrial mammal species, we used infrared sensor cameras (Game Spy I40, Moultrie Feeders, Alabaster, Alabama, USA). Cameras were fixed to tree trunks 30–150 cm above the ground, according to each site's microtopography, along various animal trails to detect as many animals as possible. We used one to three cameras simultaneously per grid. Over the study period from September 2009 to October 2010, cameras were moved to various sites within each grid to exclude local factors and to cover all seasons. We obtained camera-trapping data for 31±14 days (mean ± SD) at each site. In total, 148 camera-trapping sites (12 to 16 in each grid) were examined. The distance between camera trapping sites within grids was 1199±1261 m (mean ± SD; Table 1).

**Table 1:** Summary of camera-trapping at the study sites.

Region	Grid	No. of camera-trapping sites	Mean distance between camera-trapping sites (m)	Total camera days	No. of valid photographs*		Valid photographs per camera day	
					Indigenous species	Nonindigenous species	Indigenous species	Nonindigenous species
Boso	B1	14	720±482	450	264	92	0.59	0.20
Peninsula	B2	13	1360±1731	418	119	23	0.28	0.06
	B3	13	508±243	446	60	21	0.13	0.05
	B4	16	603±550	451	22	25	0.05	0.06
	B5	12	237±148	330	90	2	0.27	0.01
	B6	16	1539±1388	454	46	4	0.10	0.01
Tama	T1	16	748±914	567	85	5	0.15	0.01
Hills	T2	16	1762±1370	519	82	8	0.16	0.02
	T3	16	1987±1568	512	83	61	0.16	0.12
	T4	16	1795±1107	501	62	175	0.12	0.35
Total		148	1199±1261	4648	913	416	0.20	0.09

* A valid photograph is one in which a mammal species (except dogs, mice, and rats) can be identified.

Compared to methods such as capturing and direct observation, camera-trapping is an effective method with lower labor costs and fewer artificial influences on wild mammals [48]. Although baited traps (fruits, nuts, or meats) and field signs (tracks, droppings, or bark damage on trees) are quite effective for detecting target species [49], the range of habits of detectable species is limited. Alternatively, camera-trapping allows researchers to detect various mammal species comprehensively. However, camera-trapping is sensitive to animal body size and is ineffective at detecting small mammals [50]. Therefore, we excluded small mammals (e.g., Murinae and Talpidae) from the analysis. Instead, our camera-trapping data were used to study the assemblage of large and mid-sized mammals. Because all the cameras used had the same sensor sensitivity, we can compare the detected animal assemblages among study sites.

### Mammal fauna

East Asia belongs to the Palearctic ecozone, similar to Europe. Detectable large mammals (body weight ≥15.0 kg) in the studied region were Asiatic black bear (*Ursus thibetanus*), sika deer (*Cervus nippon*), wild boar (*Sus scrofa*), and Japanese serow (*Capricornis crispus*), and the mid-sized mammals (1.0 kg ≤ body weight <15.0 kg) are Japanese macaque (*Macaca fuscata*), Reeves' muntjac (*Muntiacus reevesi*), raccoon (*Procyon lotor*), Japanese badger (*Meles anakuma*), red fox, raccoon dog (*Nyctereutes procyonoides*), cat (feral, stray, or free-roaming housecats; *Felis catus*), masked palm civet (*Paguma larvata*), Japanese hare (*Lepus brachyurus*), and Japanese marten (*Martes melampus*) [25], [51], [52].

To achieve accurate estimates, we analyzed the habitat preferences of only those mammal species that occurred in at least 5 of the 148 camera-trapping sites. We excluded small mammals (body size <1.0 kg), and dogs because our cameras were not suitable for photographing small mammals, and dogs are almost always led by humans in the study area. However, we did analyze the habitats of small mammals if there was a sufficient number of photographs taken in at least 5 camera-trapping sites, because we could compare relative preference among habitats, although we could not consider absolute abundance.

Among the large and mid-sized mammals, we defined Reeves' muntjac, raccoon, masked palm civet, and cat as nonindigenous species, based on Ohdachi et al. [51]. Reeves' muntjac is native to China, and this species escaped from the zoo in southern Boso Peninsula between the 1960s and 1980s. Raccoon is native to North America, and these animals were brought to Japan as pets mainly in the 1970s. Masked palm civet is native to China and Southeastern Asia; this species is regarded as nonindigenous in Japan, although the original place and period of introduction are unknown. *F. catus* is one of the major nonindigenous species in the world, and cats have lived in Japan since before the year 800.

### Quantification of landscape and other factors

**Figure 2:**
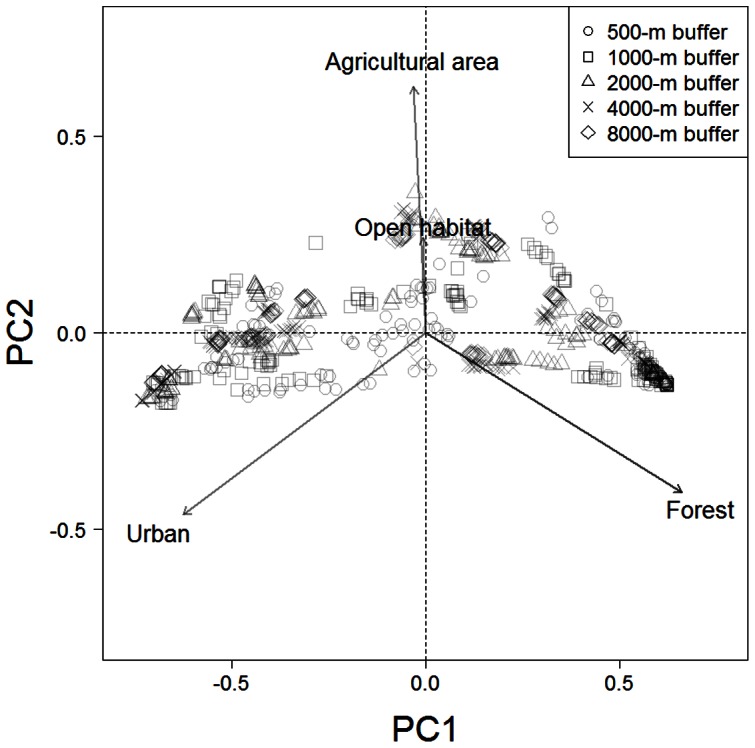
Surrounding land use of the camera-trapping sites. Principal component analysis of the camera-trapping sites was performed based on land use (forest, agricultural land, open habitat [grassland and golf course], and urban area). Land use in five buffer sizes (500-, 1000-, 2000-, 4000-, and 8000-m radius) for each site were combined into one dataset (sites × land uses), and each study site appears five times on this graph. With this analysis we were able to calculate the PC1 value, which represented the position within the urban–rural–forest landscape gradient, of a single site at various spatial scales.

**Figure 3:**
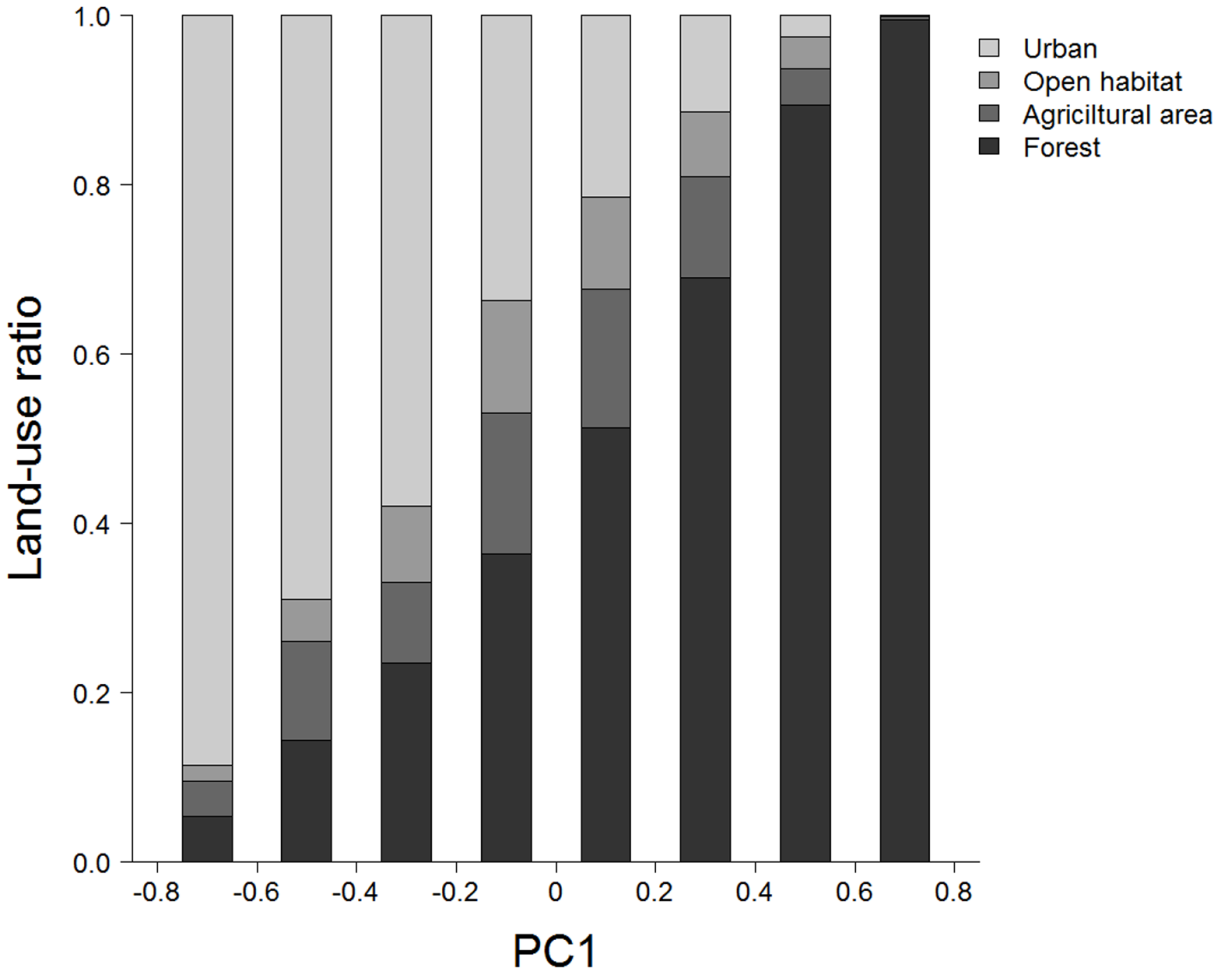
Land-use ratio along the urban–rural–forest landscape gradient. PC1 is the first component of the principal component analysis for camera-trapping sites based on land use (Figure 2).

A recent vegetation map [53] was used to quantify the landscape. To evaluate the landscape surrounding each camera-trapping site, we obtained the ratios of forest, agricultural land, open habitat (grassland and golf course), and urban area within distance *r* m (i.e., buffer size) using a vegetation map. We examined buffer sizes of 500-, 1000-, 2000-, 4000-, and 8000-m radius from each camera-trapping site to clarify the key spatial scale for each mammal species. To avoid multicollinearity, we conducted a principal component analysis (PCA) based on the land-use ratios in each buffer size class. In a preliminary PCA (Figure 2), the first axis (PC1) explained 91.4% of the total variances of the variables. A larger value of PC1 indicated much forest cover, whereas a lower PC1 value indicated urban landscape at all spatial scales (Figures 2 and 3). The second axis (PC2) represented agricultural land, but its contribution was limited (6.9%). Thus, PC1 was used to define the urban–rural–forest landscape gradient. We calculated PC1 scores for each spatial scale from the PCA results. These analyses were conducted using the princomp function of the stats package in R 2.15.0 [54].

To consider the factors affecting parameter estimates, we used topographic and seasonal factors. As a topographic factor, we calculated curvature [55] from a 10-m digital elevation model using the Spatial Analyst tool in ArcMap 9.3 (ESRI, Redlands, California, USA). Curvature is an index value of concavity and convexity: positive values indicate ridges, negative values show valleys, and zero values represent flat land. As a seasonal factor, we scored survey periods as March to August (i.e., spring–summer) (1) or September to February (i.e., autumn–winter) (0), because the activity levels of mammals in Japan are higher in spring and summer than in autumn and winter [56]. To check the collinearity among variables, we calculated the correlation coefficient among all factors (PC1 and the topographic and seasonal factors). Because the values were very low (|*r*|<0.15), we assumed that collinearity was not an issue. By considering these factors explicitly, we can distinguish the effects of local topography and seasons within the general landscape effect.

### Modeling

To analyze the effects of the landscape gradient on the distribution of mammal species, we used a logistic regression model incorporating the difference in camera-trapping days, as follows:
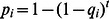
(1)

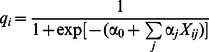
(2)where *p_i_* is the probability of occurrence at trapping site *i*, *q_i_* is the probability of occurrence per unit camera-day at trapping site *i*, *t* is the number of the camera-day, α_0_ and α*_j_* represents the regression coefficients, and *X_ij_* represents the *j*th explanatory variable at trapping site *i*. The occurrence (presence or absence) of each species at one camera site was the response variable, and PC1, PC1^2^, curvature, and season were the explanatory variables. PC1^2^ was considered to detect a bell-shaped mammal distribution, with the highest occurrence at the intermediate zone of the landscape gradient (i.e., rural or suburban landscape). We performed a best-subset model selection procedure based on Akaike's information criterion (AIC) for variable selection in order to select the important factors. Likelihood maximization for modeling was conducted using the nlm function of the stats package in R 2.15.0 [54]. The relative importance of the lowest AIC model was evaluated by Akaike weight [57].

Thtough this model construction, we obtained the lowest AIC model for each buffer size of each species. Next, we employed a model selection procedure based on AIC to detect the key spatial scale. We obtained the best model for each species by comparison among the lowest AIC models of each buffer size. We also evaluated the difference between the AIC of the best model and other spatial scale models (ΔAIC). If ΔAIC for a given scale was small, then that spatial scale had a similar goodness-of-fit as the best scale, and the critical scale was not necessarily clear [57]. Thus, we did not adopt a unique model, but rather several spatial scales of ΔAIC<2.

As a preliminary analysis, we checked spatial autocorrelation of our data, because spatial autocorrelation may lead to misleading parameter estimates [58]. As an index of the spatial autocorrelation, we constructed Moran's *I* correlograms using residuals of equation 1. Because Moran's *I* for all lag distances in each model were low (|*I*|<0.2), we assumed that there were no spatial autocorrelation [59], [60]. These analyses were conducted using the moran function of the spdep package in R 2.15.0 [54].

### Detection of dominant species

We defined three landscapes using PC1: urban landscape (PC1<−0.4), rural landscape (−0.4≤PC1<0.4), and forest landscape (PC1≥0.4). Because animal body weight affects detectability by camera traps [50], we should consider the species body weight as well as frequency of camera detection. We divided mammal species into large (body weight ≥15.0 kg) or mid-sized (1.0 kg ≤ body weight <15.0 kg) body weight category, and compared species within each category. There was a large gap in body weight between macaque (the largest in mid-size species) and serow (the smallest in large-size species), and 15 kg threshold was in this gap. According to Tobler et al. [50], 15 kg weight was intermediate detectability. In each landscape in each body weight category, dominant mammal species were identified based on detection frequency.

## Results

**Figure 4:**
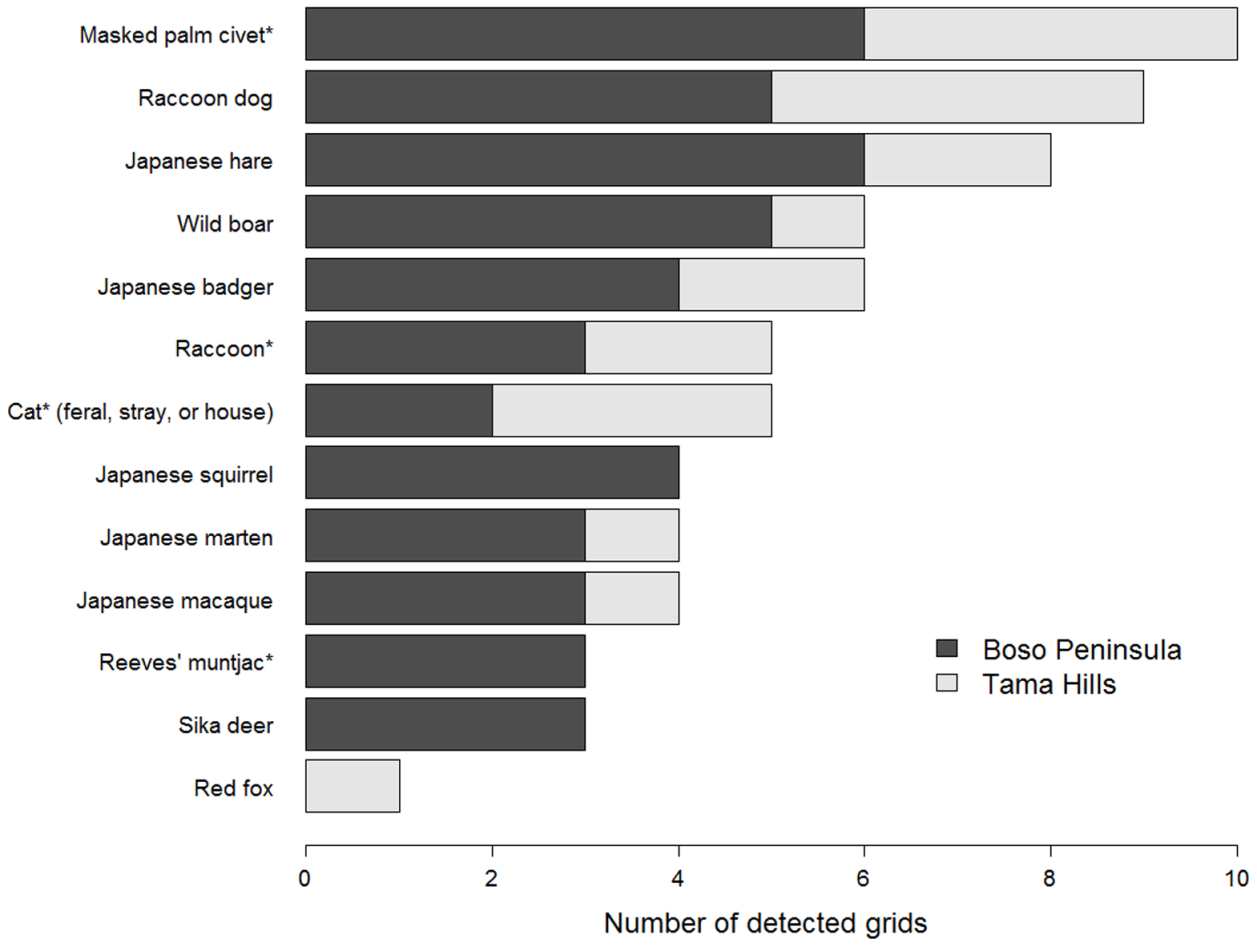
Number of grids in which each mammal species was found. An asterisk indicates a nonindigenous species.

We used 1329 valid photographs (2902 invalid photographs) of mammal species (except dogs, mice, and rats) for habitat analysis (Table 1). We observed 12 large and mid-sized mammal species at the 148 camera-trapping sites (Figure 4). Asiatic black bear and Japanese serow were not detected in our study area, and the habitats of red fox were not analyzed due to insufficient detected sites. Among the small species detected, Japanese squirrel was included in the habitat analysis because we obtained a sufficient number of photographs.

### Species distribution along the urban–rural–forest gradient

**Figure 5:**
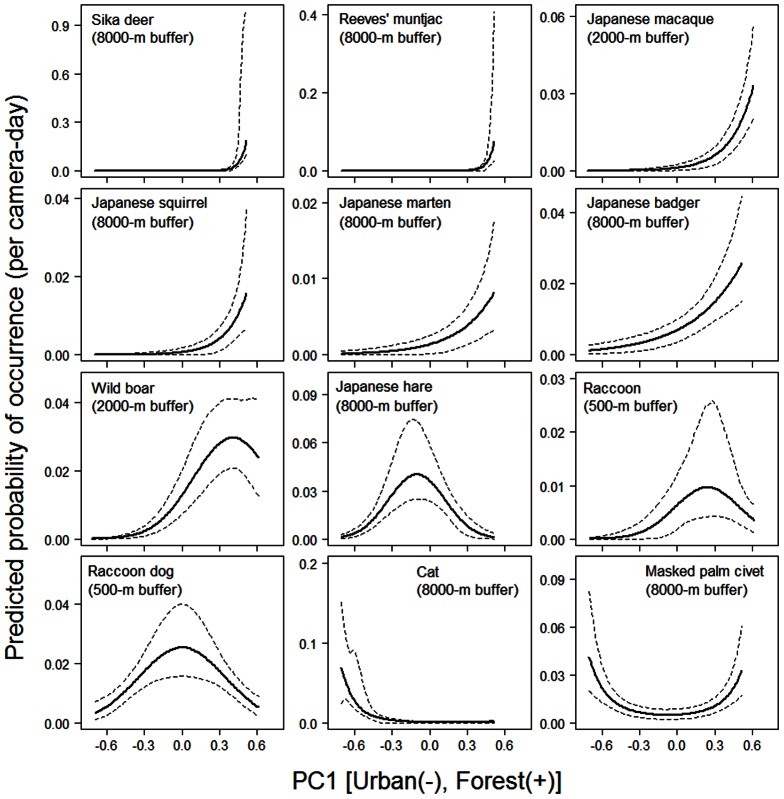
Mammal species occurrence along the urban–rural–forest landscape gradient. Regression curves (Table 2) and 95% confidence intervals (a 2000 iteration bootstrap, shown as dashed lines) are shown. PC1 is the first component of the principal component analysis for camera-trapping sites based on land use (Figure 2); a larger PC1 value indicates forest landscape, a smaller value indicates urban landscape, and an intermediate value indicates rural landscape (Figure 3). In this analysis we assumed average values of regression coefficients for season and local topography (Table 2). The best buffer size models are shown.

In the habitat model, PC1 (the linear delegate of the urban–rural–forest gradient) was selected in the best model of all species, and we depicted the distribution of each mammal species along PC1. The sika deer, Reeves' muntjac, Japanese macaque, Japanese squirrel, Japanese marten, and Japanese badger had higher occurrence probabilities at larger PC1 values, indicating that they preferred the deep forest landscape (Figure 5). PC1^2^ was significant for the wild boar, Japanese hare, raccoon, and raccoon dog (Table 2), and these mammals showed bell-shaped distributions along the landscape gradient. Among these species, wild boar had a peak of occurrence close to the forest landscape, whereas the peaks were in intermediate landscape for the others. Cats (feral, stray, or free-roaming housecats) were found in urban areas, and masked palm civets occurred in both urban and forest landscapes.

**Table 2:** The lowest AIC model for predicting the occurrence of each species for each buffer size (500-, 1000-, 2000-, 4000-, and 8000-m radius).

Species	Buffer size	Coefficient					AIC	Akaike weight	ΔAIC
		Intercept	PC1	PC1^2^	Curvature	Season			
Sika deer	500 m	−16.7	21.2				76.5	0.32	41.4
	1000 m	−22.9	32.6				53.3	0.23	18.2
	2000 m	−10.1	12.2				43.1	0.19	8.0
	4000 m	−9.9	13.4				39.1	0.25	4.0
	8000 m	−12.8	21.8				35.1	0.24	0
Reeves' muntjac	500 m	−21.5	28.3				56.4	0.23	21.4
	1000 m	−24.1	32.3			1.3	45.3	0.36	10.3
	2000 m	−12.0	13.0			1.2	38.2	0.31	3.2
	4000 m	−12.1	14.4			1.4	35.6	0.36	0.6
	8000 m	−14.5	21.8			1.5	35.0	0.34	0
Japanese macaque	500 m	−9.3	8.7				93.2	0.38	9.4
	1000 m	−9.0	8.7				86.7	0.37	2.9
	2000 m	−6.8	5.6				83.85	0.34	0
	4000 m	−6.5	5.7				83.90	0.35	0.05
	8000 m	−6.7	6.8				85.8	0.37	2.0
Japanese squirrel	500 m	−8.1	5.1				71.8	0.31	6.4
	1000 m	−7.6	4.6				71.1	0.34	5.7
	2000 m	−7.1	4.3				68.3	0.35	2.9
	4000 m	−7.1	4.9				66.1	0.34	0.7
	8000 m	−7.3	6.2				65.4	0.35	0
Japanese marten	500 m	−7.4	4.1		0.15		66.4	0.25	3.0
	1000 m	−7.0	3.0				65.2	0.24	1.8
	2000 m	−6.7	3.0				64.1	0.26	0.7
	4000 m	−6.6	3.3				63.4	0.25	0
	8000 m	−6.6	3.5				64.2	0.25	0.8
Japanese badger	500 m	−5.9	2.4			0.84	138.8	0.41	2.2
	1000 m	−5.7	2.1			0.87	139.4	0.43	2.8
	2000 m	−5.5	2.2			0.89	137.9	0.38	1.3
	4000 m	−5.4	2.3			0.90	137.1	0.36	0.5
	8000 m	−5.4	2.6			0.87	136.6	0.45	0
Wild boar	500 m	−4.7	3.9	−4.05			147.0	0.28	17.1
	1000 m	−4.4	3.9	−4.78			134.9	0.37	5.0
	2000 m	−4.3	4.2	−5.20			129.9	0.34	0
	4000 m	−4.1	3.7	−5.38			131.1	0.36	1.2
	8000 m	−4.1	3.5	−6.03			132.3	0.37	2.4
Japanese hare	500 m	−3.4	0.89	−5.92			171.5	0.30	11.9
	1000 m	−3.4		−4.93			174.3	0.37	14.7
	2000 m	−3.6		−5.72			168.7	0.27	9.1
	4000 m	−3.5	−1.3	−7.16			170.0	0.45	10.4
	8000 m	−3.3	−2.1	−9.99			159.6	0.52	0
Raccoon	500 m	−5.1	3.5	−7.28			102.1	0.40	0
	1000 m	−5.0		−2.65			107.1	0.17	5.0
	2000 m	−4.9		−4.43			103.5	0.31	1.4
	4000 m	−5.0		−4.00			105.1	0.27	3.0
	8000 m	−5.6					107.4	0.18	5.3
Raccoon dog	500 m	−3.6		−4.24	−0.10		156.5	0.29	0
	1000 m	−3.8	−1.0	−2.51	−0.09		162.0	0.29	5.5
	2000 m	−4.2	−1.3	−1.74	−0.09		163.3	0.21	6.8
	4000 m	−4.5	−1.2		−0.10		161.3	0.22	4.8
	8000 m	−4.6	−1.4		−0.10		158.5	0.27	2.0
Cat	500 m	−5.4	−3.2				102.7	0.32	19.9
	1000 m	−6.1	−4.2				92.4	0.32	9.6
	2000 m	−7.0	−3.3	4.03			84.5	0.31	1.7
	4000 m	−7.1	−3.5	3.99			84.0	0.30	1.2
	8000 m	−7.1	−3.1	4.65			82.8	0.29	0
Masked palm civet	500 m	−5.0		1.96		0.52	188.9	0.19	11.4
	1000 m	–5.3		2.81		0.52	186.7	0.26	9.2
	2000 m	−5.1		2.62		0.51	185.1	0.26	7.6
	4000 m	−5.2		3.25		0.50	181.8	0.23	4.3
	8000 m	−5.5	0.83	5.50	−0.07	0.51	177.5	0.22	0

The best model is the model with the lowest AIC in each species. Explanatory variables were selected by the AIC of the logistic regression for each combination. Akaike weight is the relative importance of the lowest AIC model in each buffer size. ΔAIC is the difference between AIC of the best model and the given spatial scale model.

### Dominant species in assemblages

**Figure 6:**
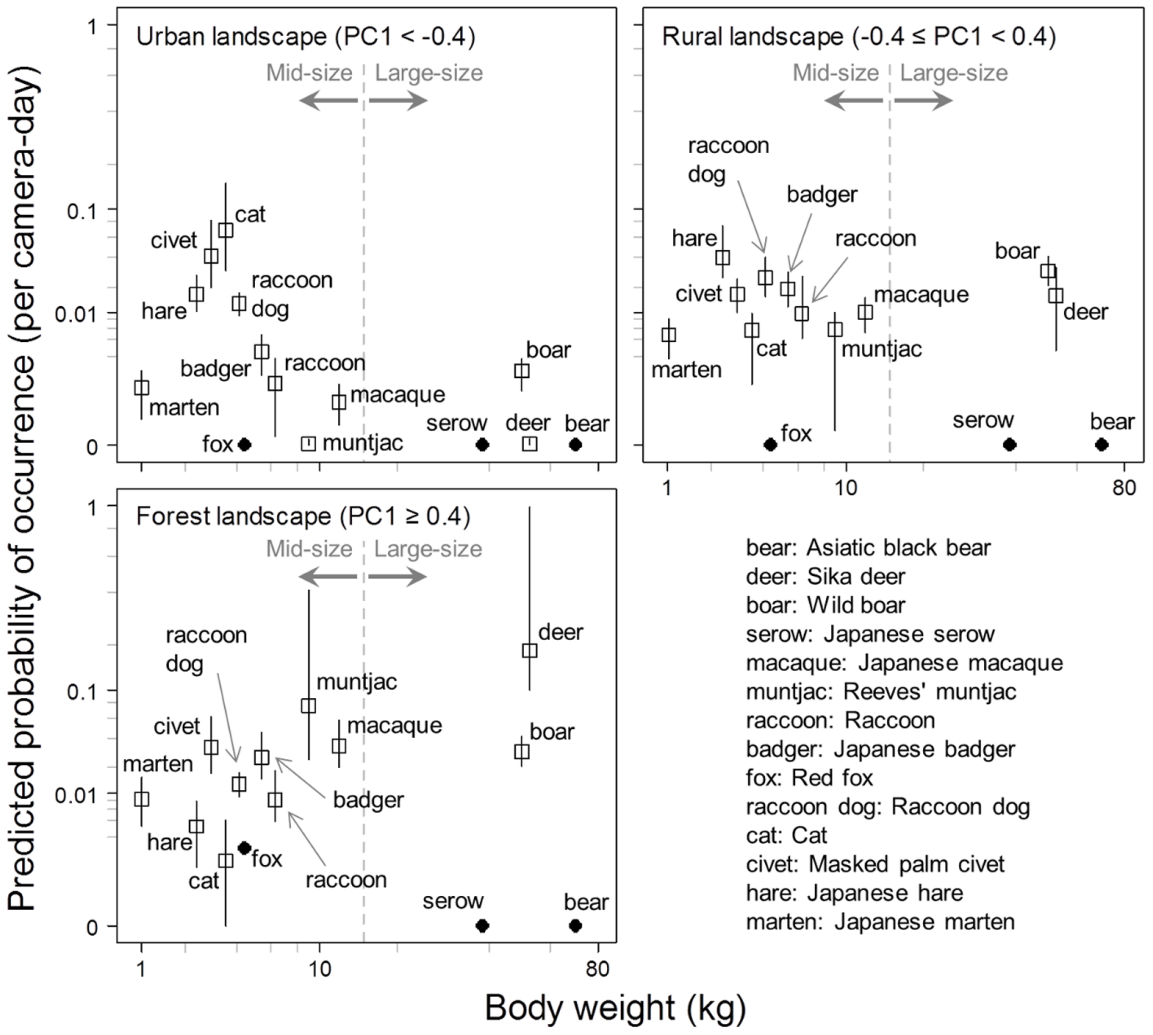
Mammal assemblages in the three landscapes. Mammal occurrence probabilities (by one camera within one day) are shown against species body weight [51], [52]. Open square indicates predicted value by the best model (Figure 5), and vertical bar indicates the range of the 95% confidence interval. In each graph, the position of the Asiatic black bear, Japanese serow, and red fox are the observed value; we could not obtain regression models of these species due to their rarity.

Among the large mammal species (i.e., Asiatic black bear, sika deer, wild boar, and Japanese serow), sika deer dominated in the forest landscape, whereas wild boar was dominant in the rural and urban landscapes (Figure 6). Among mid-sized species (i.e. Reeves' muntjac, Japanese macaque, Japanese marten, Japanese badger, Japanese hare, raccoon, raccoon dog, cat, masked palm civet, and red fox), the dominant species in the forest landscape were Reeves' muntjac, Japanese macaque, masked palm civet, and Japanese badger. Japanese hare and raccoon dog were dominant in the rural landscape, followed by Japanese badger and masked palm civet. The urban mammal assemblage was dominated by cat and masked palm civet. Thus, masked palm civet was common in both urban and forest landscapes. Red fox was not detected in the urban landscape.

### Topography and seasons

In addition to the urban–rural–forest gradient, land curvature was selected in the best model for the raccoon dog and masked palm civet (Table 2). These species were often photographed near valley bottoms. Season was selected in the best model for the Reeves' muntjac, Japanese badger, and masked palm civet, which were most often photographed during the spring and summer.

### Spatial scale

The key spatial scale was a buffer size of more than 4000 m for 8 of the 12 species analyzed: sika deer, Reeves' muntjac, Japanese squirrel, Japanese marten, Japanese badger, Japanese hare, cat, and masked palm civet (Table 2). Although the key spatial scale of Japanese macaque and wild boar were 2000 m, the difference of AIC between the 2000-m and 4000-m models were low (ΔAIC<2; Table 2), suggesting that 4000 m buffer size is nearly equally important for these species. Conversely, the key spatial scales for the raccoon and raccoon dog were 500 m.

### Nonindigenous mammals

On average, nonindigenous mammals were observed 0.09 times per camera-day, compared to 0.20 times for indigenous species (Table 1). Nonindigenous mammals were major constituents of mammal assemblages around the Tokyo metropolitan area. Among the four nonindigenous species photographed, the masked palm civet was one of the most common species in this region, occurring in all survey grids from urban to deep forest (Figure 4).

## Discussion

The landscape gradient was the most important factor affecting the distribution of wild mammals in the study area (Table 2). Based on our quantitative modeling, we can predict the mammal assemblage in a given landscape. For mammals preferring forest, a landscape scale larger than 4000 m in radius (Table 2) was crucial. Mammals respond rapidly to landscape change [44], [61], and such large patches of forest landscape should be maintained in the Tokyo metropolitan area. Conservation and management plans for wild mammals, especially forest species, should be considered in the context of the large-scale landscape [62], [63]. However, small green spaces will also be important for mammal conservation in the urban landscape in which large forest patches have disappeared, because the key spatial scale of raccoon dogs, one of the main indigenous species in the urban landscape, was narrow. Conservation plans must consider the spatial scale of management according to the degree of urbanization.

### Mammal assemblages along the urban–rural–forest landscape gradient

In general, sightings of many mammal species decreased from forest landscapes to urban cores, although some species preferred anthropogenic landscapes. Large species (deer and boar) and some mid-sized species (marten, badger, and macaque) generally dominated the mammal assemblage of the forest landscape, and this result agrees with individual species distributions reported in various studies [24], [25], [64]–[68]. The raccoon, raccoon dog, and Japanese hare had bell-shaped distributions along the landscape gradient and dominated the intermediate rural landscape (Figure 5). The occurrence of such species in intermediate landscapes has been reported for raccoons in North America [69] and for raccoon dog and Japanese hare in the Tama Hills and Kanto mountain region [25], although dominance in the assemblages was not clear in these previous studies. The domestic cat is usually found in urban landscapes (Figure 5) [24].

### Variations in distribution patterns

For some species, distribution patterns along the urban–rural–forest landscape gradient differ among regions. For example, red foxes are common in urban areas in cool-temperate zones, both in London, England [31], and Sapporo, Japan [32]. We recorded red foxes at only two camera-trapping sites in the forest landscape (T1 grid, Figure 4), and red fox was not detected in the urban landscape (Figure 6). Thus, future research should be conducted in various regions (e.g., different climate and land-use histories) to clarify the generalized distribution pattern of mammal assemblages.

Taxonomically similar species sometimes show different distribution patterns. For example, Japanese squirrel occurred only in the forest landscape (Figure 5; see also [25], [70]), but its congener *Sciurus carolinensis* is often found in urban parks in North America [71]. Such differences also occur within species. Some forest mammals, such as the sika deer, wild boar, and Japanese badger, sometimes live in urban or suburban environments at religious shrines and in residential areas if feeding by people suppresses their fear of humans [72]–[74].

### Factors determining mammal distribution along the urban–rural–forest landscape gradient

The availability of food and shelter and the avoidance of humans are important factors in the landscape preferences of mammals [68], [69], [71], [75]. In Japan, forest landscapes provide a good place to hide from humans for many mammals, and this may be a major reason for the high mammal diversity in the forest landscape.

Artificial grasslands and crops in the rural landscape provide good plant foods for many herbivorous and omnivorous animals. Even species that prefer forests (e.g., sika deer, Japanese macaque, Japanese badger, and wild boar) utilize the crops in agricultural fields close to forest edges [66], [76]–[79]. The raccoon and raccoon dog also eats crops [29], [80]–[82]. The Japanese hare often feeds on artificial grassland and at forest edges in anthropogenic landscapes [66], [83], [84].

Garbage in urban landscapes contains food of animal origin, which can be consumed by carnivorous and omnivorous animals. Raccoons and raccoon dogs often feed on garbage in urban areas [29], [80]–[82]. Cats usually reproduce only around human residential areas due to their dependency on anthropogenic food resources [25], [85], [86], although they have been reported to become naturalized in non-urban open habitats with seabird breeding sites [87]. These findings suggest that open habitat with the availability of food of animal origin is a key factor in the distribution of cats.

Urban and rural landscapes provide anthropogenic shelters, such as abandoned houses and roof spaces. Raccoons and masked palm civets can use these sites for dens and shelters [51], although forests also provide den sites [88].

An animal's fear or habituation to humans may affect its habitat preferences [89]. The fear of humans might be a key mechanism underlying the distribution of large forest mammals, who could use plant food resources in the rural landscape (sika deer and wild boar), to avoid anthropogenic landscapes. However, people are usually not aware of the existence of nocturnal mid-sized or small mammals, such as masked palm civets, even if these animals live around homes in residential areas.

### Spatial scale

The raccoon and raccoon dog had small key spatial scales (Table 2). A circle of 500-m radius (78.5 ha) appears to correspond to the home range sizes of females in suburban areas (raccoon: 81–100 ha [81], raccoon dog: 61 ha [90]). Even if available habitats are small in urban or suburban areas, raccoons and raccoon dogs can use anthropogenic shelters, consume garbage, and avoid humans due to their nocturnal behavior and body size. While raccoons and raccoon dogs prefer the intermediate landscape, they both can exist to some extent in urban landscape (PC1<−0.4) and forest landscape (PC1≥0.4) (Figures 6). They may be able to pass through urban and forest landscapes as they migrate,, and reproduce in small suitable habitats in these landscapes.

Although forest species require a large spatial scale, the 4000-m radius (5024 ha) was far larger than the maximum home range size (approximately 1000 ha) of each species [51]. Therefore, the large buffer size may reflect the amount of habitat required for population maintenance [91], rather than the home range. Alternatively, forest species may prefer to be a long distance from anthropogenic landscapes to avoid contact with humans. Individuals of these species were scarcely found in anthropogenic landscapes (PC1<−0.4) (Figures 5), and the whole population should inhabit the forest landscape.

Cats (feral, stray, or free-roaming housecats) depend on humans, which likely affected their common occurrence in large residential areas and their preference for large-scale urban landscapes. The masked palm civet showed no clear habitat preference, and further research is needed to clarify the habitat requirements and spatial scale of this species.

### Effect of range expansion of nonindigenous mammals on gradient analysis

We recorded four nonindigenous mammals (Figure 4). Among these, the masked palm civet, raccoon, and cat have almost completed their geographical range expansion in the study area [92], [93]. Reeves' muntjac is a forest-adapted small deer that is expanding its geographical range on the Boso Peninsula [94]. In England, Reeves' muntjac tolerates human disturbance and acclimates to both traffic and people [95], suggesting that this species might invade fragmented forests in the urban landscape in Japan. Thus, at this point our findings regarding Reeves' muntjac are tentative. For more accurate evaluation, it will be necessary to study this species in other regions where its range expansion has ended.

### Comparison of mammal assemblages along urban–rural–forest landscape gradients

We quantified mid-sized to large mammals along an urban–rural–forest landscape gradient using unbaited camera-trapping in warm-temperate East Asia. Although many studies of urban mammals have been conducted in North America, Europe and Australia [33], this study was the first quantitative analysis of the effects of urbanization on mammal assemblages. Comparisons of mammal assemblages in regions of the world with various climates and different urban structures reflecting various human cultures are needed clarify the general pattern of human impact on wild mammal assemblages.
